# Dataset on usage and engagement patterns for Facebook Live sellers in Thailand

**DOI:** 10.1016/j.dib.2020.105661

**Published:** 2020-05-06

**Authors:** Nassim Dehouche

**Affiliations:** Business Administration Division, Mahidol University International College

**Keywords:** Social Media Marketing, Customer Engagement, Direct Selling, Principal Component Analysis

## Abstract

This article describes a Comma Separated Values (CSV) dataset consisting of 7050 Facebook posts of various types (text, deferred and live videos, images). These posts were extracted from the Facebook pages of 10 Thai fashion and cosmetics retail sellers from March 2012, to June 2018. The dataset was collected via the Facebook API, and anonymized in compliance with the Facebook Platform Policy for Developers [1]. For each Facebook post, the dataset records the resulting engagement metrics comprising shares, comments, and emoji reactions within which we distinguish traditional “likes” from recently introduced emoji reactions, that are “love”, “wow”, “haha”, “sad” and “angry”. This dataset could serve as a basis for research on customer engagement with the novel sales channel that is Facebook Live, through comparative studies with other forms of content (text, deferred videos, and images), as well as the statistical analysis of the seasonality of engagement and outlier posts.

Specifications table

**Table:** 

Subject	Marketing
Specific subject area	Social Commerce
Type of data	Table
	Charts
How data were acquired	Facebook's developers API
Data format	Raw CSV dataset
	Descriptive statistics of raw dataset
Parameters for data collection	Facebook API accessed using a Python script
Description of data collection	Malhotra, Malhotra, and See 2013 [1]
Data source location	Bangkok, Thailand
Data accessibility	Repository name: UCI Machine Learning Repository
	Data identification number: Facebook+Live+Sellers+in+Thailand
	Direct URL to data:
	https://archive.ics.uci.edu/ml/machine-learning-databases/00488/Live.csv
Related research article	Apiradee Wongkitrungrueng, Nassim Dehouche, and Nuttapol Assarut (2020). Live streaming commerce from the seller's perspective: implications for online relationship marketing. Journal of Marketing Management, Special issue on the Future of Technology in Marketing, In Press. https://doi.org/10.1080/0267257X.2020.1748895

## Value of the data

• The dataset could serve as a basis for correlation analysis, and principal component analysis on customer engagement in social commerce using a novel sales channel that is video streaming.
• Researchers and practitioners in marketing
• The data can be further used to characterize exceptionally performing posts that would be statistical outliers in terms of engagement metrics
• Live streaming commerce is very well developed in Thailand. Indeed, the country tops the world ranking for the proportion of live streaming domestic viewers [4]. Moreover, Thailand has the World's highest proportion of shoppers buying directly from social media [5] and is considered the most advanced market in conversational commerce whereby people purchase items from businesses via messaging platforms [6].

## 1 Data description

Before the advent of live streaming, statistical studies of customer engagement associated with Facebook posts of different types [2,7,8,9], considered datasets of status updates, links, videos, and photos with the latter being exclusively of the deferred type. The common observed pattern is that photos were the most commonly used medium and typically generated the most likes and comments, followed by videos. This hierarchy has been observed not only for private brand pages [2,7,8], but also for posts on cancer information from a public research organization [9].

In addition to traditional types of posts, the dataset described in the present paper includes live videos. For each individual post (rows), the columns of the dataset record the type of posts, their date, and engagement metrics comprising shares, comments, and emoji reactions [2] within which we distinguish traditional “likes” from recently introduced emoji reactions, that are “love”, “wow”, “haha”, “sad” and “angry”, reflecting more varied sentiments than the more neutral “like” [3]. Descriptive statistics of the engagement metrics per post, for the Facebook pages of the 10 sellers considered in the dataset, are presented in Table 1. For each seller, this table presents the mean, standard deviation and maximum value of the considered engagement metrics.

**Table 1:** Descriptive statistics of engagement metrics for each Facebook seller in the Dataset.

Page #	Start Row	End Row	Statistic	Comment	Share	Like	Love	Wow	Haha	Sad	Angry
			Mean	48.44	5.11	346.49	3.28	0.38	0.11	0.17	0.02
1	2	2636	SD	588.36	33.23	605.85	12.66	1.36	0.57	1.67	0.22
			Max	20990.00	1260.00	4710.00	234.00	21.00	8.00	51.00	6.00
			Mean	180.83	22.28	85.26	8.67	0.63	0.42	0.15	0.08
2	2637	3848	SD	714.79	75.68	143.08	22.84	1.76	1.92	0.88	0.93
			Max	12003.00	856.00	1744.00	225.00	26.00	40.00	23.00	31.00
			Mean	1100.06	181.78	390.58	42.17	4.70	4.48	0.50	0.51
3	3849	3973	SD	2257.18	379.58	327.48	76.92	7.00	13.84	1.15	1.46
			Max	9452.00	1636.00	2344.00	282.00	57.00	102.00	8.00	12.00
			Mean	358.09	35.54	86.23	15.09	0.39	0.66	0.14	0.13
4	3974	4029	SD	425.78	43.96	84.93	16.43	0.71	1.15	0.44	0.38
			Max	1734.00	247.00	497.00	55.00	3.00	5.00	2.00	2.00
			Mean	0.86	3.25	2.46	0.11	0.01	0.01	0.00	0.01
5	4030	4224	SD	8.33	28.78	4.80	0.65	0.10	0.14	0.00	0.07
			Max	91.00	322.00	54.00	7.00	1.00	2.00	0.00	1.00
			Mean	28.03	20.89	14.84	2.17	0.11	0.14	0.02	0.04
6	4225	4479	SD	96.54	68.38	36.95	7.08	0.47	0.64	0.12	0.22
			Max	860.00	356.00	259.00	49.00	4.00	5.00	1.00	2.00
			Mean	599.13	233.64	699.19	91.88	18.29	4.80	0.94	0.77
7	4480	4731	SD	833.34	388.37	495.92	137.32	41.49	14.84	2.91	1.38
			Max	6174.00	3424.00	2293.00	657.00	278.00	157.00	37.00	8.00
			Mean	47.83	22.92	32.75	5.81	0.11	0.19	0.02	0.01
8	4732	5135	SD	147.32	63.21	31.10	18.47	0.39	0.78	0.16	0.11
			Max	779.00	304.00	186.00	106.00	3.00	8.00	2.00	1.00
			Mean	273.36	81.48	143.81	25.92	0.94	0.77	0.28	0.18
9	5136	6273	SD	501.04	128.87	381.03	39.47	2.60	1.46	2.21	0.94
			Max	3800.00	757.00	4241.00	198.00	23.00	12.00	46.00	19.00
			Mean	756.60	64.48	114.04	11.41	1.26	1.70	0.55	0.21
10	6274	7051	SD	1780.94	144.01	140.10	27.44	1.97	4.40	1.44	0.87
			Max	17404.00	913.00	1917.00	220.00	14.00	43.00	14.00	10.00

**Fig. 1:**
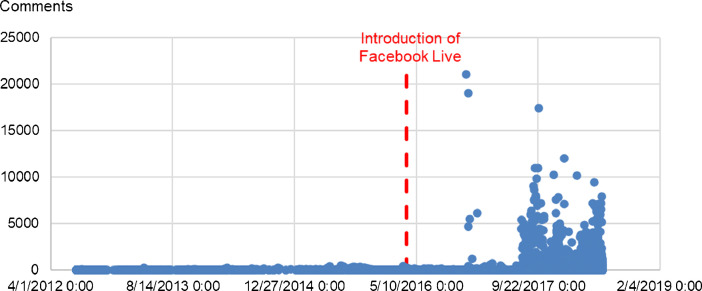
Number of Comments over time for the 10 Facebook pages in the dataset.

**Fig. 2:**
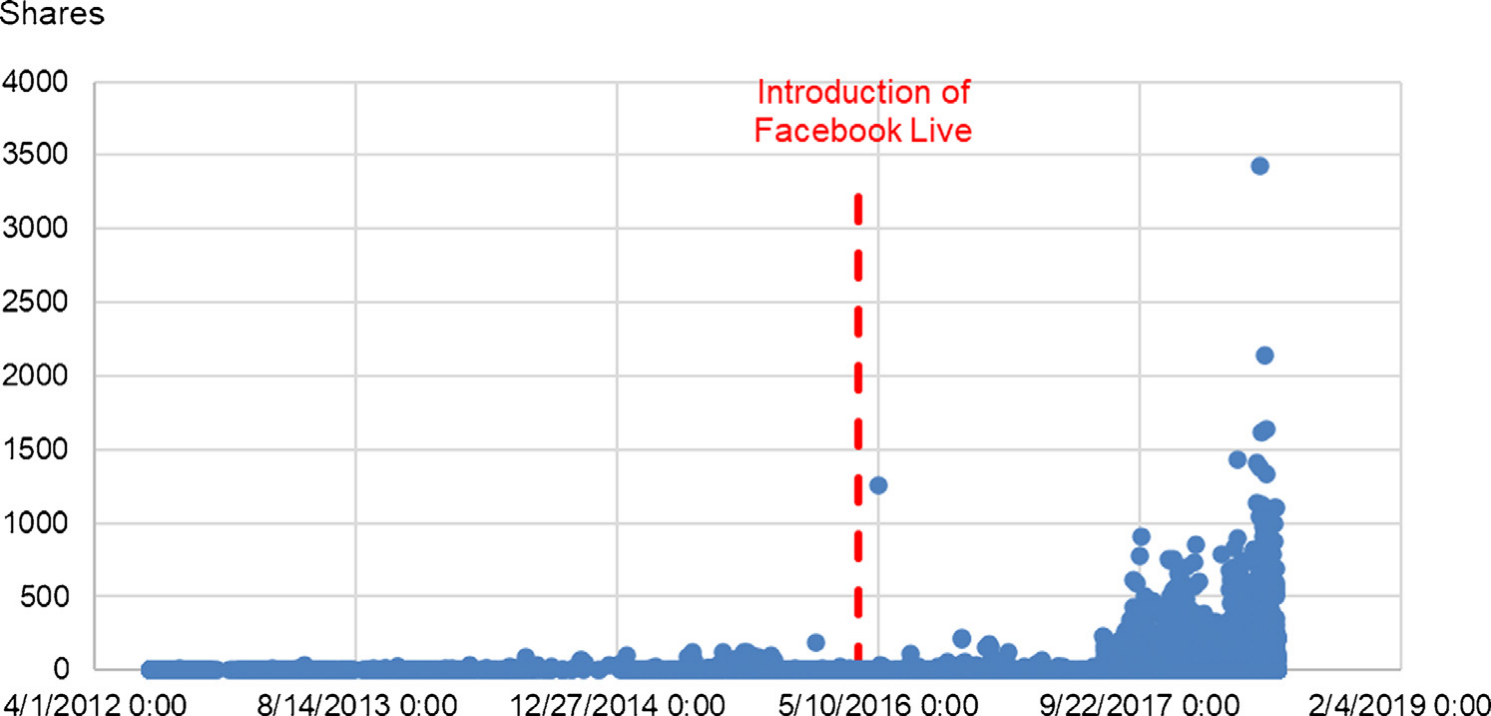
Number of Shares over time for the 10 Facebook pages in the dataset.

**Fig. 3:**
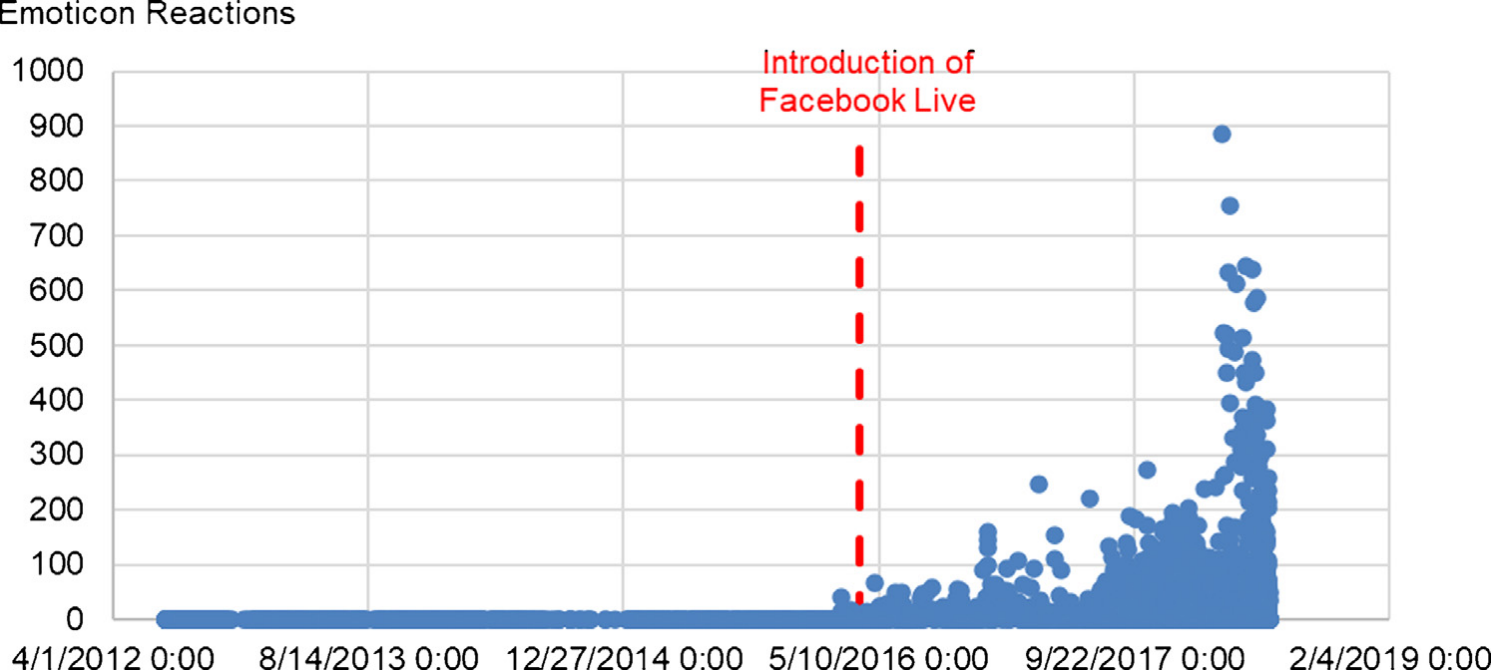
Number of Emoticon Reactions over time for the 10 Facebook pages in the dataset.

**Fig. 4:**
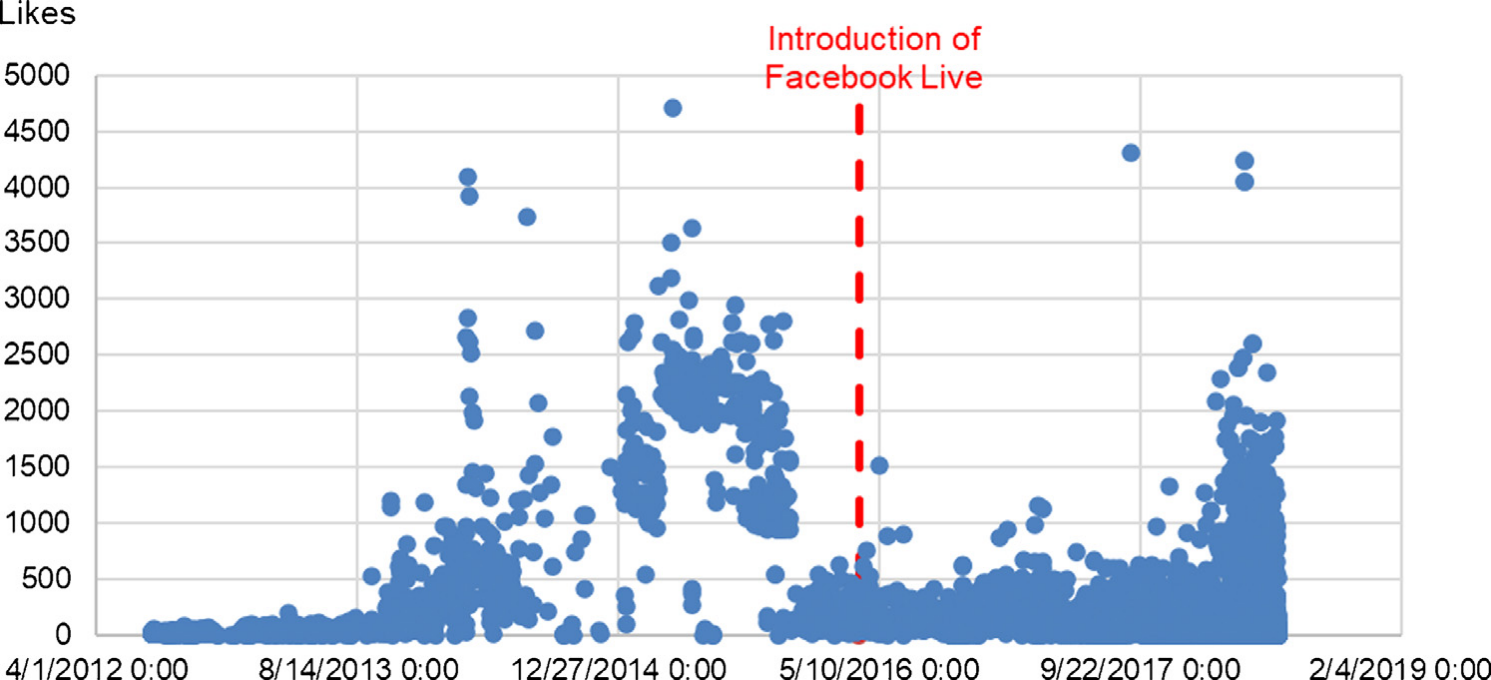
Number of Likes Reactions over time for the 10 Facebook pages in the dataset.

Individual pages considered in the dataset exhibit a wide range of values for engagement metrics, but also three main forms of interaction with their content. Indeed, we can observe that for some pages the primary form of engagement is through likes (e.g. Seller 1), when for other it can be shares (e.g. Seller 5), or likes (e.g. Seller 3). The more recently introduced emoticon reactions (“love”, “wow”, “haha”, “sad” and “angry”) exhibit lower values overall because of their unavailability for posts prior to March 2016. However, the dataset shows that live videos (introduced around the same time, in April 2016) generate a high number of ‘love’. In fact, the proposed dataset suggests that the introduction of Facebook Live videos drastically changed the statistical distribution of all engagement metrics, for all types of posts, and had a profound effect on the way followers interact with content. This can be observed in the dataset by studying the evolution of these metrics as a time-series. Fig. 1, Fig. 2, Fig. 3, Fig. 4 present graphical representations of such time-series for Comments, Shares, Emoticon Reactions, and Likes, respectively.

In these Figures, we can observe dramatically higher averages and maxima for the first three engagement metrics, before and after the introduction of Facebook Live. However, Likes do not appear to undergo the same changes.

The influence of Facebook Live on engagement metrics can be qualitatively explained by marketing theory. Indeed, it suggests stronger feelings and bonding between sellers and viewers that ties with previous findings concerning vividness and interactivity. The two factors of vividness and interactivity are commonly used as a basis for studying the user responses to different forms of online content. Both Pletikosa Cvijikj and Michahelles [7] and Luarn et al. [8] explain the higher engagement generated by photos with these two dimensions, as classically defined by Steuer [10]. They see vividness as “the extent to which a brand post stimulates various senses ”, whereas interactivity is “the degree to which users can influence the form and content of the media environment”. Pletikosa Cvijikj and Michahelles [7] conclusion is that “vividness increases, while interactivity decreases the level of engagement over moderator posts, making photos the most appealing post media type” and “providing entertaining and informative content significantly increases the level of engagement”. Luarn et al. [8] find that “using social posts is likely to elicit comments and encourage the interaction of users”.

In view of these findings, live videos represent a qualitative leap in terms of vividness, where content is as close to real life as online content delivered through a screen can be. The interactivity of live videos is however, limited to some extent and individual viewers do not have much influence over the content (as opposed e.g. to links that have users click, fill forms and follow certain paths in the sitemap of a website). This live video medium also allows live sellers to have a real-time control over the content shared. A live video can be simultaneously entertaining, social, and informative, in reaction to the feedback of the mass of viewers. Therefore, the proposed dataset can serve as a basis for comparative studies on engagement with live videos versus older forms of social media posting and potentially refine marketers’ understanding of the impact of vividness and interactivity on customer engagement.

In addition to reproducing the results of the related research article [11], which investigated the question through Principal Component Analysis, future studies relying on this dataset could attempt to investigate the influence of Facebook Live on engagement, from a time perspective. Indeed, the posts in the dataset being time-stamped, a potentially fruitful line of research could investigate how the observed increase in engagement built up over time following the introduction of Facebook Live, as well as the seasonality of engagement metrics (i.e. which hours in a day, days in a week, months in a year, see more or less engagement?).

## 2 Experimental design, materials, and methods

Facebook pages were selected based on their number of followers and activity, using the Facebook Live Map tools. Data were collected through a Python script that makes queries to the Facebook API using the URL pattern, in which “Pagename” is the name of the page of the seller, “StartDate” and “EndDate”, respectively the date of the first ever post by a page and the current date and “Token”, our access token to the Facebook API.

https://graph.facebook.com/v2.9/”Pagename”/posts/?fields=message,link,permalinkurl,createdtime,type,name,id,comments.limit(0).summary(true),shares,likes.limit(0).summary(true),reactions.limit(0).summary(true)&until=”StartDate”&since=”EndDate”&limit=100&accesstoken=”Token”

## Conflict of Interest

The author declares that he has no known competing financial interests or personal relationships which have, or could be perceived to have, influenced the work reported in this article.

## Appendix Supplementary materials

Image, application 1
